# Kinematically aligned robotic total knee arthroplasty for post-traumatic femoral recurvatum with hardware retention: a case report

**DOI:** 10.1093/jscr/rjaf983

**Published:** 2025-12-13

**Authors:** Danil Chernov, Nicholas Frappa, Thomas Listopadzki, Matthew Alben, Morgan Dillon, Alexander Kovacs, Sridhar R Rachala

**Affiliations:** Jacobs School of Medicine and Biomedical Sciences, 955 Main St. Buffalo, NY 14203, United States; Jacobs School of Medicine and Biomedical Sciences, 955 Main St. Buffalo, NY 14203, United States; Department of Orthopaedics and Sports Medicine, 462 Grider St. Buffalo, NY 14215, United States; Department of Orthopaedics and Sports Medicine, 462 Grider St. Buffalo, NY 14215, United States; Jacobs School of Medicine and Biomedical Sciences, 955 Main St. Buffalo, NY 14203, United States; Department of Orthopaedics and Sports Medicine, 462 Grider St. Buffalo, NY 14215, United States; Department of Orthopaedics and Sports Medicine, 462 Grider St. Buffalo, NY 14215, United States

**Keywords:** total knee arthroplasty, kinematic alignment, robotic-assisted surgery, extra-articular deformity, hardware retention

## Abstract

We describe a 58-year-old male with severe posttraumatic osteoarthritis and distal femoral recurvatum deformity following adolescent fracture fixation. Preoperative imaging demonstrated a healed malunion stabilized by a lateral locking plate and tricompartmental osteoarthritis with 12° recurvatum. A single-stage robotic-assisted kinematically aligned total knee arthroplasty was performed using cementless cruciate-retaining components while retaining the femoral plate. Careful preoperative planning and intraoperative adjustment of bone resections enabled restoration of joint line orientation, balanced soft tissues, and stable press-fit fixation without stems or osteotomy. At two-year follow-up, the patient reported excellent function and pain-free mobility, with outcome scores including KOOS JR 91.98, Oxford Knee Score 47, and Forgotten Joint Score 100. This case highlights the feasibility of robotic-assisted, hardware-retaining, cementless TKA in the setting of complex extra-articular deformity, avoiding staged hardware removal or constrained implants while achieving durable clinical outcomes.

## Introduction

Extra-articular deformities of the femur or tibia can complicate total knee arthroplasty (TKA) by distorting the limb’s anatomy and alignment [1]. Traditionally, managing a deformed femur or tibia during TKA might require either a corrective osteotomy or the use of constrained implants to circumvent the deformity [2]. Performing an osteotomy adds risk of nonunion whereas using long-stemmed, constrained components require more bone resection that can compromise implant longevity [3, 4]. Additionally, the presence of retained hardware from prior fracture fixation often obstructs intramedullary guides and may require staged hardware removal [5].

Kinematic alignment (KA) with intraoperative dynamic gap balancing has emerged as an alternative alignment strategy aiming to restore the knee’s native anatomy and laxity [6] Kinematic alignment in TKA seeks to resurface the joint by replicating the patient’s native pre-arthritic joint lines and kinematic axes, thereby avoiding ligament releases that are often required in mechanical alignment, which standardizes all knees to a neutral mechanical axis [7]. Robotic-assisted TKA enables this philosophy to be executed with greater precision, providing accurate pre-operative three-dimensional planning and controlled bone resections that reproduce kinematic or restricted kinematic alignment more reliably than manual techniques [7, 8]. Recent evidence also highlights the importance of carefully considering existing hardware management in these settings. A large meta-analysis found that conversion TKA carries a higher risk of periprosthetic joint infection (PJI) when hardware is removed compared to retained, and that if removal is required, a staged approach is associated with a lower PJI risk than concurrent removal [9].

We present a case of severe post traumatic knee osteoarthritis (OA) with a pronounced extra-articular recurvatum deformity managed successfully with a single-stage robotic-assisted KA-TKA. The femoral and tibial hardware were retained and uncemented unconstrained components were implanted. We describe the surgical strategy and compare it to traditional methods, highlighting the benefits of hardware retention and modern technology in complex arthroplasty.

## Case report

A 58-year-old male with a history of left distal femur fracture and subsequent open reduction and internal fixation (ORIF) in late adolescence developed progressively worsening left knee pain and deformity. During clinical examination, he had a stiff knee with range of motion from 10–90° with a flexion contracture of 10°. There was no gross coronal plane malalignment clinically, but a palpable posterior femoral bow was noted.

**Figure 1:**
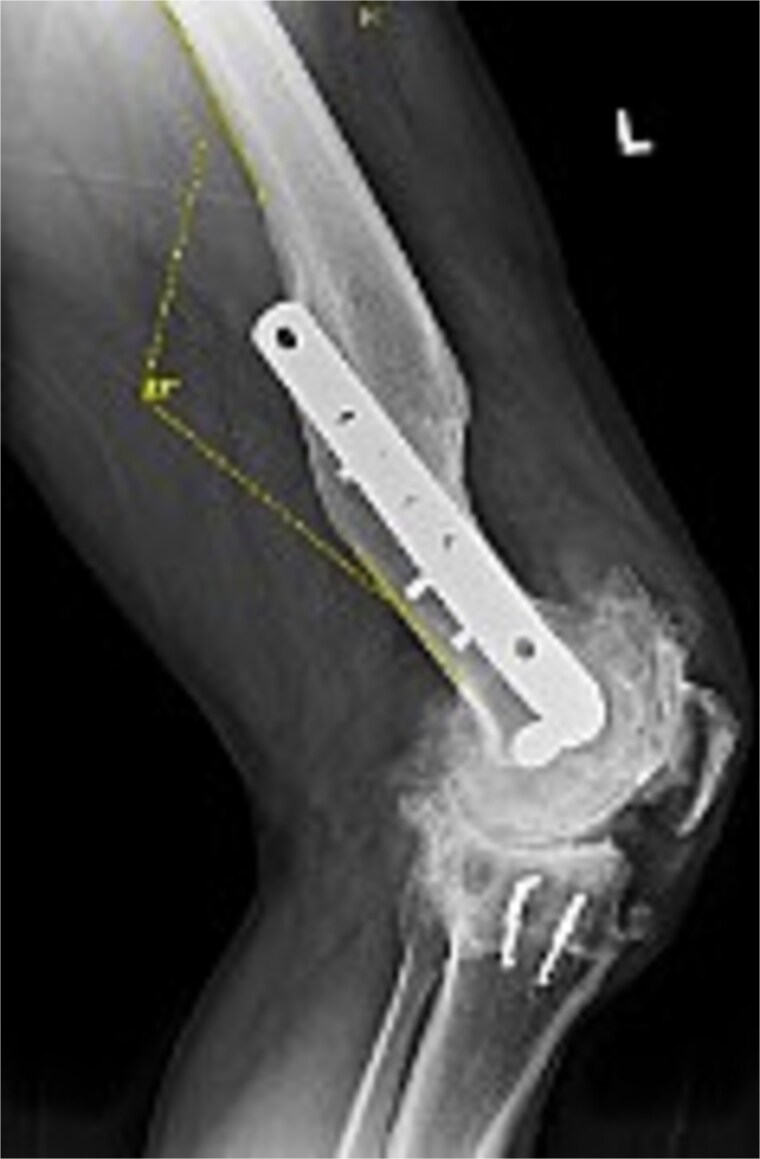
Preoperative lateral radiograph of the left knee and distal femur. The pre-existing lateral plate and screws are seen along the anterior femoral cortex. A pronounced recurvatum malunion of the distal femur is evident measured at 12°. The knee joint shows severe tricompartmental osteoarthritic changes with joint space obliteration and osteophyte formation.

**Figure 2:**
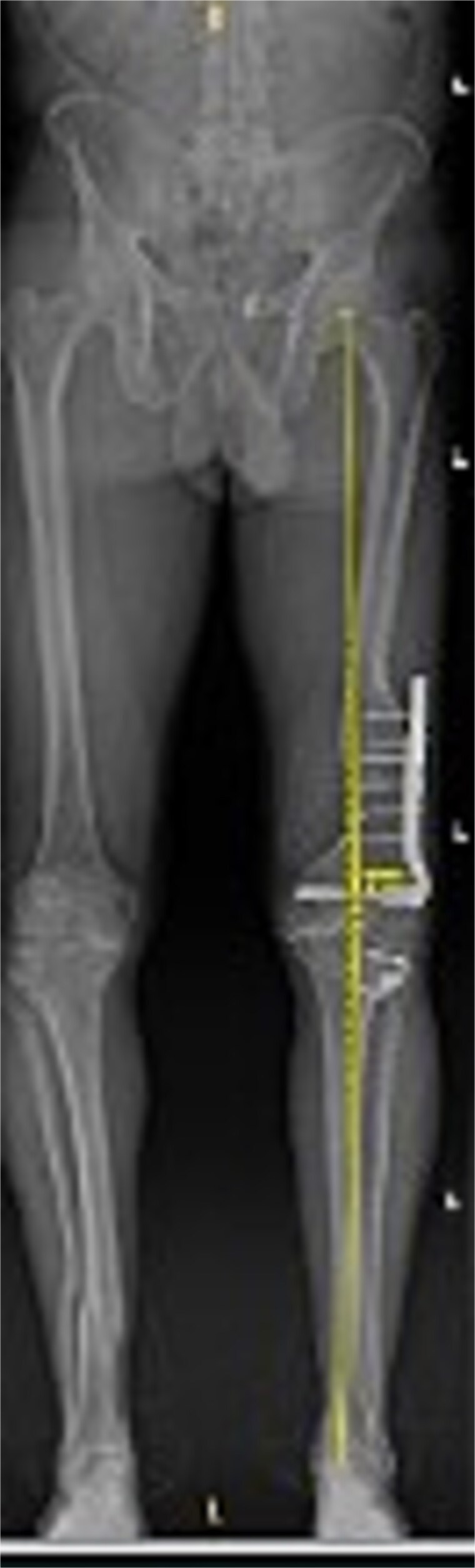
Preoperative standing AP radiograph of the patient’s lower extremities. The left femur has a lateral plate from a prior fracture. There is 3° varus alignment of the arthritic left knee. The femoral deformity is primarily in the sagittal plane, and the retained hardware is visible.

Preoperative radiographs revealed end-stage OA with osteophytes and subchondral sclerosis. Figure 1 shows the lateral view of the distal femur and knee. A healed malunion with significant recurvatum of 12° is evident, stabilized by a locking plate spanning the distal third of the femur. The plate and screws remained securely fixed, and the distal femur deformity altered the knee’s sagittal alignment with minimal impact on the coronal alignment. The AP standing long-leg radiograph (Fig. 2) demonstrated varus alignment of ~3°. The extra-articular deformity was located ~10 cm proximal to the knee joint line. No loosening of hardware or nonunion was noted. After discussing options, including staged deformity correction versus one-stage TKA, the decision was made to proceed with a single-stage TKA as the patient had a reasonable range of motion without hyperextension and a preference to avoid multiple surgeries.

### Operative technique

The procedure was performed through a standard medial parapatellar approach. A robotic-assisted TKA system (Stryker Mako™) was utilized to guide bone resection and alignment. Preoperative CT-based planning allowed detailed visualization of the deformity and enabled kinematically aligned resections. The surgical plan aimed to restore the patient’s native distal femoral joint line and posterior slope, rather than enforcing a neutral mechanical axis, thereby minimizing the risk of collateral ligament imbalance.

Intraoperatively, the distal femoral cut was adjusted slightly in flexion to account for the patient’s recurvatum deformity, ensuring an appropriate extension gap without causing component overhang. Particular attention was given to preserving posterior condylar offset. The patient’s prior femoral hardware, located anterolaterally and proximally, did not interfere with the planned resections and was retained. One of the tibial staples was removed using a bur. Soft tissue releases were deliberately minimized, limited to osteophyte removal, and a moderate posteromedial capsular release.

An uncemented cruciate-retaining prosthesis was selected, achieving stable press-fit fixation without the need for stem extensions. The patella was prepared freehand and resurfaced with an uncemented asymmetric 35-mm component. Following trialing, the knee demonstrated stability throughout the full arc of motion, with balanced ligament tension and satisfactory patellar tracking. Final components were implanted, and the wound was closed in layers.

### Postoperative outcome

**Figure 3:**
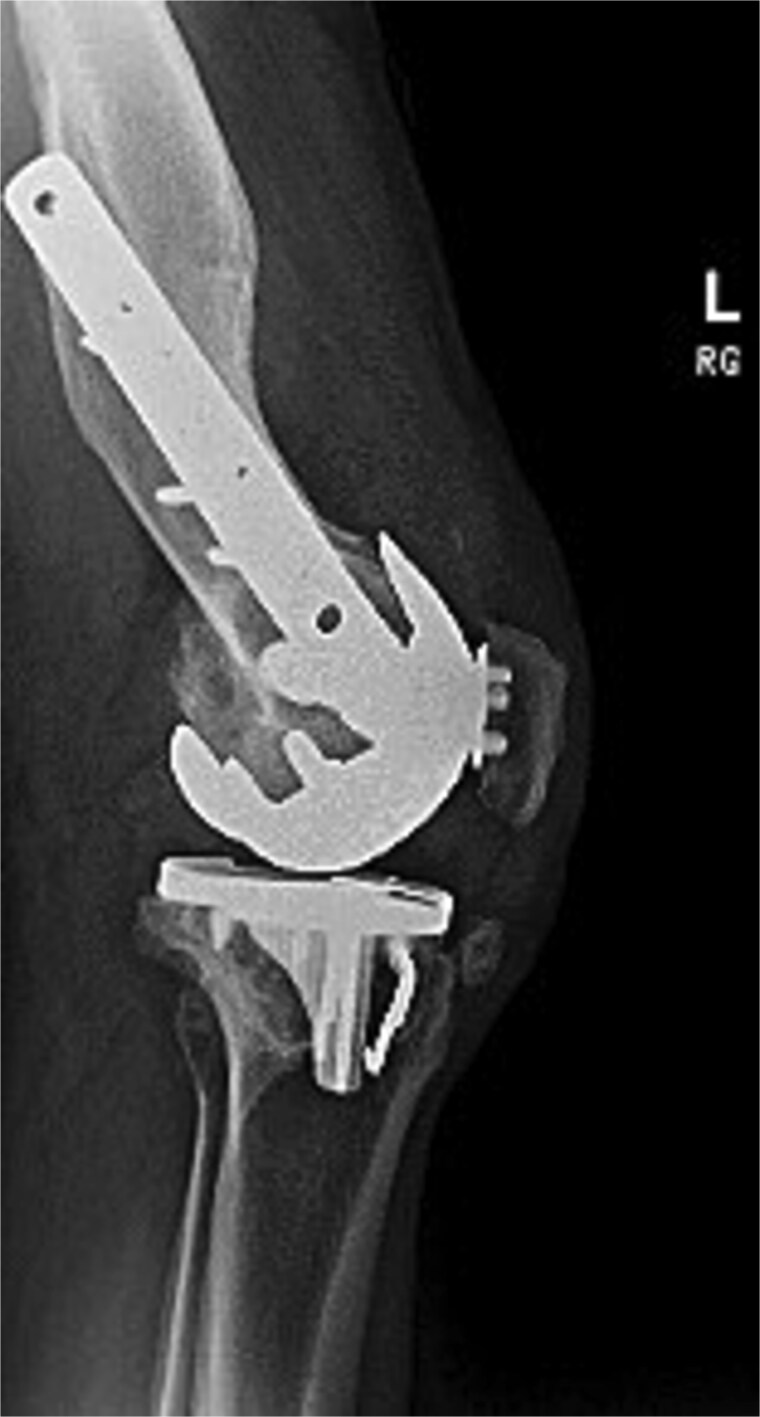
Postoperative lateral radiograph of the left knee 1 year after robotic-assisted TKA with hardware retention. The new femoral and tibial components are in place. The retained femoral plate and screws sit flush against the lateral cortex.

**Figure 4:**
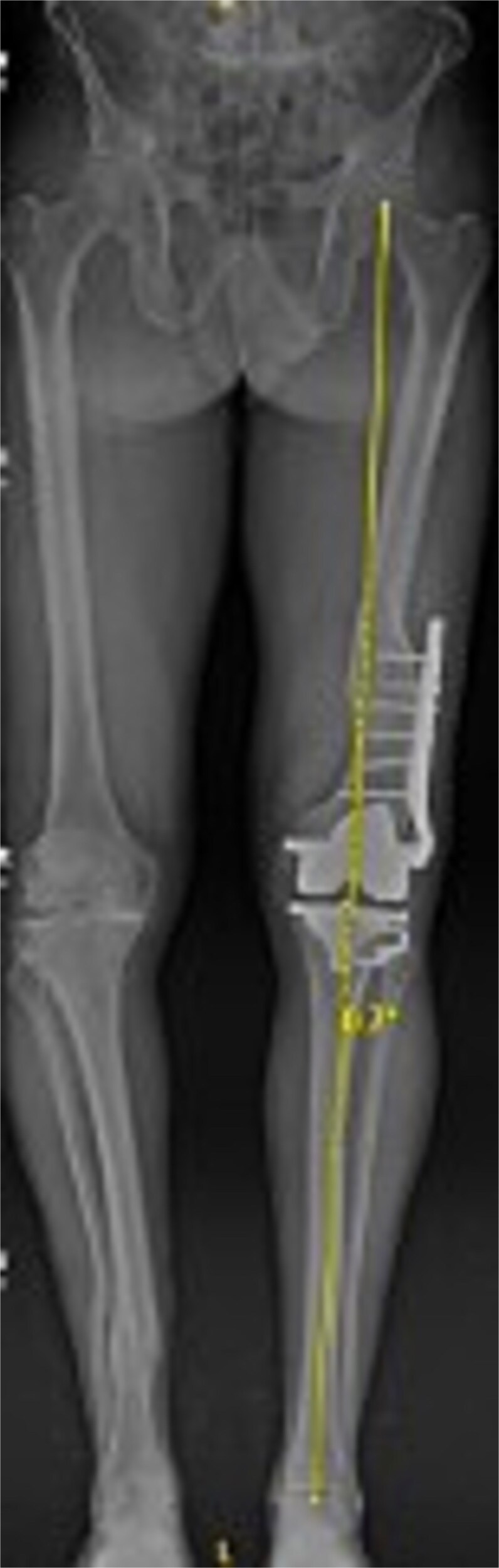
Postoperative AP bone length films 1 year after robotic-assisted TKA with hardware retention. The new femoral and tibial components are in place. The retained femoral plate is visible along the lateral cortex of the femur. Postoperative mechanical alignment of the left leg is 0.7°.

Figures 3 and 4 show a postoperative lateral and AP radiograph of the left knee. The patient was able to fully extend his knee after surgery and instructed to weight bear as tolerated until his two-week follow-up. Patient-reported outcome measures were completed at 2 year follow up (Table 1) with no reported complaints or issues at that time.

**Table 1:** Patient-reported outcome scores 2 years following total knee arthroplasty

**Outcome measure**	**Score**
KOOS, JR. (Knee Injury and Osteoarthritis Outcome Score for Joint Replacement)	91.98/100
Oxford Knee Score (OKS)	47/48
Forgotten Joint Score (FJS)	100 / 100
PROMIS – Pain Interference	Excellent
PROMIS – Mobility	Excellent
PROMIS – Global Health	Excellent

## Discussion

This case demonstrates the successful use of robotic-assisted, kinematically aligned TKA in a patient with severe posttraumatic osteoarthritis, a 12° extraarticular recurvatum deformity, and retained distal femoral hardware. Using a cementless, stemless, cruciate-retaining design, we achieved excellent 2-year clinical outcomes without the need for osteotomy or hardware removal. To our knowledge, this is the first reported case describing this technique in a single-stage primary TKA.

Previous studies have shown that navigation- or robot-assisted TKA is feasible in the presence of extra-articular deformity or retained hardware, enabling precise bone cuts when conventional intramedullary guides are obstructed [5–8]. In this case, robotic planning allowed for distal femoral resections that respected the anterior cortex and joint line orientation without requiring neutral mechanical alignment. Kinematic alignment helped restore natural soft tissue tension, avoiding the need for extensive ligament releases or constrained implants, and has been shown to better reproduce normal gait patterns compared to mechanical alignment [7]. A recent report by Alturki *et al.* [10] supports this technique, showing that robotic functional or personalized alignment in extra-articular deformity yields near-neutral postoperative alignment and improved early function.

Hardware retention has been reported as safe in select cases. Manzotti *et al.* [11] demonstrated that computer-assisted systems can enable hardware-retaining TKA without compromising alignment or increasing complications. Cerny *et al.* [5] similarly retained a distal femoral locking plate to avoid weakening the femoral shaft. Our case involved a distally positioned lateral locking plate, and given the patient's prior surgeries, we prioritized preserving existing fixation to prevent stress risers and iatrogenic fracture [12].

Implant selection in posttraumatic cases is often dictated by deformity severity, fixation challenges, and bone quality. Many surgeons favor stemmed or constrained components in this setting, but these may increase operative time, bone resection, and long-term risk of loosening or nonunion [5, 13]. In contrast, we achieved balanced flexion and extension gaps and implant stability with uncemented, unconstrained components. While cemented fixation remains the standard in posttraumatic TKA, recent data suggest that modern cementless press-fit implants offer comparable survivorship and lower aseptic loosening in younger, healthier patients [14]. Among prior cases involving deformity or hardware retention, none have reported the use of cementless, stemless fixation. Our report may represent the first case combining all of these elements in a single-stage surgery.

In summary, this case reinforces the growing body of evidence that robotic-assisted, kinematically aligned TKA can be safely and effectively performed in patients with extra-articular deformity and retained hardware. With precise planning, limited soft-tissue balancing, and appropriate implant selection, a single-stage, hardware-retaining, cementless primary TKA is both feasible and durable. At 2 years, our patient reported favourable PROMs and remained symptom-free, highlighting the viability of this strategy.
